# Piezo1 mechanosensitive channels: what are they and why are they important

**DOI:** 10.1007/s12551-019-00584-5

**Published:** 2019-09-07

**Authors:** Pietro Ridone, Massimo Vassalli, Boris Martinac

**Affiliations:** 1 Mechanosensory Biophysics Laboratory, Victor Chang Cardiac Research Institute, 405 Liverpool St, Darlinghurst, NSW 2010 Australia; 2 School of Biotechnology and Biomolecular Science, University of New South Wales, Kensington, NSW 2052 Australia; 3 Institute of Biophysics, National Research Council, Genoa, Italy; 4 St Vincent’s Clinical School, University of New South Wales, Darlinghurst, NSW 2010 Australia

**Keywords:** Lipid bilayer, Force-from-lipids, Force-from-filament, Transbilayer pressure profile, Patch clamp, Liposome reconstitution

## Abstract

Mechanosensitive (MS) ion channels are integral membrane proteins which play a crucial role in fast signaling during mechanosensory transduction processes in living cells. They are ubiquitous and old in the evolutionary sense, given their presence in cells from all three kingdoms of life found on Earth, including bacterial, archaeal, and eukaryotic organisms. As molecular transducers of mechanical force, MS channels are activated by mechanical stimuli exerted on cellular membranes, upon which they rapidly and efficiently convert these stimuli into electrical, osmotic, and/or chemical intracellular signals. Most of what we know about the gating mechanisms of MS channels comes from the work carried out on bacterial channels. However, recent progress resulting from identification and structural information of eukaryotic K2P-type TREK and TRAAK as well as Piezo1 and Piezo2 MS channels has greatly contributed to our understanding of the common biophysical principles underlying the gating mechanism and evolutionary origins of these fascinating membrane proteins. Using Piezo1 channels as an example, we briefly describe in this review what we have learned about their biophysics, physiological functions, and potential roles in “mechanopathologies.”

## Introduction

Living cells have throughout evolution learned to cope with and respond to various mechanical stimuli by possessing a range of proteins associated with their cellular envelope, including cell wall, cytoskeleton (CSK), extracellular matrix (ECM), and membrane proteins. Among them, mechanosensitive (MS) ion channels are force-sensing integral membrane proteins, whose function is tightly linked to the lipid bilayer of cell membranes. MS channels operate on a millisecond time scale and are thus usually found at the very origin of cellular signaling pathways involved in mechanosensory transduction processes. Given the omnipresence of mechanical stimuli acting on the cell membrane, it is important to note that the function of integral membrane proteins other than MS channels, such as GPCRs or membrane-associated phospholipases, can also be modulated by the application of mechanical force (Martinac and Cox 2017; Storch et al. 2012; Lehtonen and Kinnunen 1995). MS ion channels, however, present an excellent example of coupling membrane proteins and their structural dynamics to the mechanics of the cell membrane. Although a large number of MS channels have over the last 30 odd years been identified at the molecular level in organisms encompassing all types of life forms from bacteria to humans, the 3D structure of only several of them has been determined by X-ray crystallography or more recently by cryo-electron microscopy (cryo-EM) (Bass et al. 2002; Chang et al. 1998; Dong et al. 2015; Brohawn et al. 2012; Ge et al. 2015; Guo and Mackinnon 2017; Zhao et al. 2018; Saotome et al. 2018; Murthy et al. 2018; Wang et al. 2019). Their function has been studied in a great variety of cells and tissues using a range of experimental and computational approaches, as described in many excellent reviews, some of which are listed here (Hamill and Martinac 2001; Sachs 2010; Martinac 2011; Kung 2005; Kocer 2015; Gillespie and Walker 2001; Chalfie 2009; Martinac and Cox 2017). By focusing on Piezo1 ion channels, we provide in this article a brief overview on the following: (i) what has been learned about how MS channels detect mechanical stimuli, (ii) how they convert these stimuli into structural transitions between the closed and open channel structures, and (iii) how different cellular components may be involved in these structural changes.

## Diversity of mechanosensitive channels and importance of Piezo1

MS ion channels are pore-forming membrane proteins that gate in response to mechanical stimuli exerted on the cell membrane. By switching between the closed and open conformations, these channels allow ions and other solutes to flow across the cellular membranes (Martinac and Cox 2017; Hamill and Martinac 2001). They have been shown to play a key role in many physiological processes associated with mechanosensory transduction, including osmoregulation in plants, fungi, and bacteria as well as hearing, touch, proprioception, and blood flow regulation in mammalian cells (Martinac 2004; Ranade et al. 2015; HonorÉ et al. 2015). At present, MS channels are considered to be a major class of mechanosensory membrane proteins acting as molecular transducers of mechanical stimuli on a millisecond time scale and converting them into electrical and/or chemical intracellular signals bypassing millions of ions and solutes when they are open (Martinac 2013). A good example is given by members of the bacterial MscL and MscS channel families that by opening upon activation by mechanical force quickly release osmolytes from bacterial cells, which become swollen due to the increase in the turgor pressure. By responding to membrane tension resulting from the increase in turgor, MscL- and MscS-like channels protect the bacterial cells from bursting upon a hypo-osmotic shock (Martinac 2011; Hamill and Martinac 2001).

**Fig. 1:**
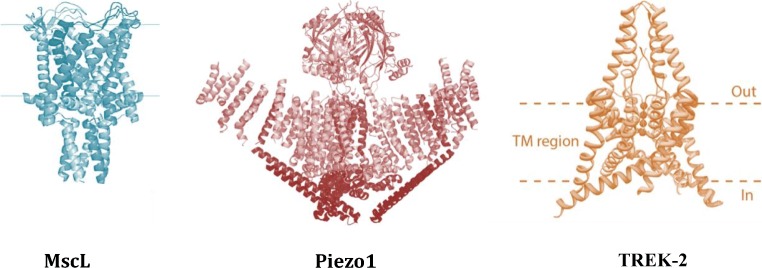
Examples of truly mechanically activated ion channels. MscL, Piezo1, and TREK-2 are activated and controlled by mechanical force over their whole dynamic range from the fully closed to the fully open state. The 3D X-ray structure of MscL from *Mycobacterium tuberculosis* shows the channel homopentamer in the closed state (adapted from Chang et al. (1998)). The Piezo1 structure determined by cryo-EM shows the first low-resolution structure of the trimeric channel in its closed conformation (adopted from Ge et al. (2015)). The higher resolution structure has later been determined by several laboratories (Guo 2017; Zhao et al. 2018; Saotome et al. 2018). Crystal structure of the human TREK-2 channel, a member of the K2P family which forms dimers. Each monomer adds two-pore loops to the structure to end up with pseudo-tetrameric assembly (Dong et al. 2015). One of the structural characteristics of the truly mechanically gated channels is recently resolved lipid-binding domains, similar to the MscL N-terminus (Bavi et al. 2016). Such lipid-binding structural domains were identified in the MscS-like channels, K2P family of channels (TREK/TRAAK), and most recently Piezo1 ion channels (Bavi et al. 2017a, b)

To define an MS ion channel as a truly mechanically gated channel, its activity must be controlled over the whole dynamic range from the fully closed to the fully open state by mechanical force alone (Fig. 1).

The forces that lie within the range experienced for these channels in vivo correspond to membrane tensions of up to ~ 25 mN/m, which roughly corresponds to the average lytic tension of the lipid bilayer of cellular membranes (Bavi et al. 2014; Nomura et al. 2012). It is worth noting that, besides the true MS channels, there are also ion channels that can be modulated by mechanical stimuli but their activity is primarily dependent on factors other than mechanical force such as membrane potential or ligand concentration (Martinac and Cox 2017).

Given that a large proportion of our knowledge on MS channels comes from “non-specialized” cells, the necessary and sufficient criteria that have generally been accepted as a proof of the true physiologically relevant mechanosensitivity of an ion channel are as follows:

1. The ion channel is required for cellular mechanotransduction and its removal abolishes the mechanosensory response.
2. Structural mutations introduced into the channel alter its functional properties and consequently, they affect the mechanosensory cellular response.
3. Heterologous expression of the channel gives rise to robust mechanosensory responses.

It is, nevertheless, important to remember that although very useful for experimental characterization of MS channels, these basic criteria cannot account for the biological complexities arising from the structural plasticity of many ion channel families and functional redundancy characteristic of many physiological systems. This can make it difficult to unequivocally determine the molecular origins of mechanosensitive currents encountered in a large variety of living cells. The role in physiology/ical processes of many MS channels is beyond doubt as attested by the example of Piezo1 channel, which this review is focusing on. Piezo1, a member of the newly discovered family of MS channels (Coste et al. 2010), is a truly mechanosensitive channel as will be shown and discussed later in this review. Together with Piezo2, it is one of the long-sought principal types of molecular force sensors in mammalian cells. It enables cells to decode various physical stimuli and presents an essential component of many mechanosensory processes, including vascular development and erythrocyte volume homeostasis in humans. Despite only recently being identified, Piezo1 gene variants have been linked to several human pathologies such as hereditary xerocytosis (Fotiou et al. 2015) and generalized lymphatic dysplasia (Lukacs et al. 2015). Thus, the medical importance of Piezo1, as well as other MS channels, has been recognized given the role that abnormal MS channel activity plays in the pathophysiology of many diseases. These diseases are collectively referred to as mechanochannelopathies, which will be dealt in more detail later in this review.

## A quest for the general mechanism of gating mechanosensitive channels by mechanical force

**Fig. 2:**
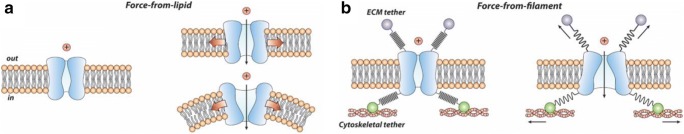
Gating paradigms of MS channels. The two main generally accepted gating paradigms of MS channels are defined according to the cell membrane components (lipid bilayer (**a**) or ECM/CSK (**b**)) transmitting the activation force directly to MS channels

The exact mechanism(s) of MS channel gating are currently largely unknown due to the relatively young age of this research area in mechano-biology/physiology. Despite the inherent complexities in mechanical force transduction in mammalian systems, there are two main gating paradigms that have generally been accepted to apply to gating of MS channels in both prokaryotic and eukaryotic cells. One of the paradigms is known as the *force-from-lipids* (Martinac et al. 1990; Kung 2005; Teng et al. 2015) and the other one as the *force-from-filament* (Fig. 2) (Chalfie 2009; Katta et al. 2015).

## Force-from-lipids or force-from-filament?

The force-from-lipids (FFL) gating paradigm implies that mechanical force activates MS channels through the lipid bilayer alone with no requirement for other cellular components. It has been proposed about 30 years ago in conjunction with studies on bacterial MS channels (Martinac et al. 1990). Unlike bacterial cells, animal and human cells do not have the rigid cell wall, but they possess instead membrane invaginations in the form of ruffles, folds, and microvilli. These invaginations contribute excess membrane area, which by unfolding upon stress protect the cell membrane from excessive strain. In addition, the membrane of mammalian cells is supported by ECM as external scaffolding, whereas CSK is strengthening the membrane from the intracellular side.

For most of the time, it was believed that only prokaryotic (bacterial and archaeal) MS channels were gated according to the FFL paradigm because bacteria have only a rudimentary CSK, from which modern mammalian CSK might have evolved (Barry and Gitai 2011), and rely largely on the cell wall to protect their fragile cytoplasmic membrane. Importantly, it has recently been shown that mammalian MS channels, including K2P-type TREK-1, TREK-2, and TRAAK (Berrier et al. 2013; Brohawn et al. 2014b; Brohawn et al. 2014a) ion channels as well as Piezo1 (Cox et al. 2016a; Cox et al. 2016b; Syeda et al. 2016) and OSCA channels (Murthy et al. 2018), are also gated by the FFL mechanism.

## Transbilayer pressure profile and gating of MS channels

**Fig. 3:**
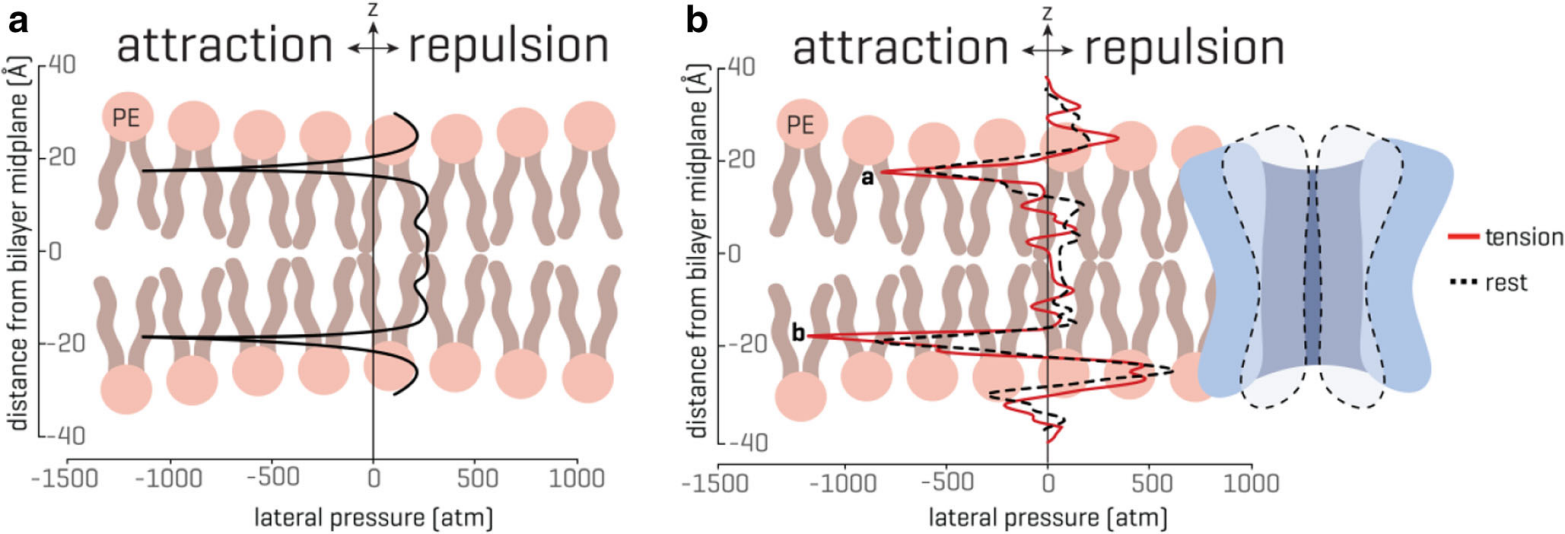
Transbilayer pressure profile. The transbilayer pressure profile is largely inhomogeneous across the bilayer thickness, which originates from the amphipathic nature of the lipid molecules and the presence of water. (**A**) An idealized symmetrical lipid bilayer. The transbilayer pressure profile shows characteristic negative peaks at the water-lipid interface (~ 1000 atm) and repulsive positive peaks (~ 300 atm) in the headgroup and tail region. The z indicates the bilayer thickness direction. (**B**) In the presence of a membrane protein, the pressure profile in a symmetrical lipid bilayer becomes noticeably asymmetric. Peak **a** and peak **b** represent the rise in the pressure profile at the lipid solvent interface (modified from Cox et al. 2016b; for more details see also Bavi et al. 2016)

The distinction between the FFL and force-from-filaments (FFF) mechanism of gating MS channels by mechanical force seems to be a false dichotomy, which is confusing rather than clarifying the molecular principles. Thus, the question about a general mechanism that may reduce the two paradigms to a single basic principle of MS channel mechanosensitivity remains to be addressed. As a possibility, the transbilayer pressure profile (Fig. 3) appears as an ideal candidate for reducing the two gating mechanisms to the FFL paradigm only.

*Transbilayer pressure profile* (Fig. 3A) refers to a strong anisotropic internal stress in the lipid bilayer resulting from the amphipathic nature of the membrane lipids, which drives the bilayer self-assembly in aqueous environment by minimizing the exposure of the phospholipid aliphatic chains to water (Cantor 1999). This is because exposure of the hydrophobic lipid tails to water is energetically expensive and thus unfavorable. The hydrophilic lipid head groups, which are either polar or charged, repel each other and are pulled together due to hydrophobicity of the phospholipid tails to prevent water molecules from entering the lipid bilayer (Fig. 3A). This results in a strong attraction of phospholipid molecules at the water-lipid interface corresponding to negative pressure peaks of up to 1000 atm. Towards the middle of the lipid bilayer, there is an increase in mobility of the lipid tails, which depends on the saturation of the lipids forming the bilayer. For mono-unsaturated phospholipid bilayers, this results in the pressure inside the bilayer of about 300 atm (Gullingsrud and Schulten 2004; Ridone et al. 2018). These stress/pressure distributions within the bilayer have been mostly estimated using a variety of computational approaches. Recently, the transbilayer pressure profile of lipid bilayers made of mono- and poly-unsaturated phospholipids was experimentally determined using NMR spectroscopy, which largely confirmed the results from the computational studies. Concomitantly with patch clamp experiments, the NMR results demonstrated that changes in transbilayer pressure profile were directly related to modulation of MS channel gating (Ridone et al. 2018) in agreement with previous findings showing that the stress heterogeneity along the bilayer thickness alters the conformation of membrane proteins (Cantor 1999). Reciprocally, membrane proteins, including MS channels, can redistribute the transbilayer pressure profile causing a noticeable asymmetry in the pressure profile, which is characterized by a reduction of the pressure peaks in the tails and significant asymmetry between the peaks at the lipid solvent interface (Fig. 3B) (Cantor 1999; Lundbaek et al. 2010). Thus, when an MS channel becomes displaced in the bilayer by stretching the membrane, it is likely that the change in the bilayer pressure profile asymmetry can lead to the channel opening, as recently demonstrated for the TREK-2 ion channel (Clausen et al. 2017). In addition, the pressure profile is highly susceptible to physical and chemical stimuli (Cantor 1999), which seems to explain the effect of the insertion of amphipathic compounds into a single leaflet of the lipid bilayer, including conical lipids such as lysophosphatidylcholine (LPC) or phosphatidic acid (PA), on the activity of various MS channels, including Piezo1 (Syeda et al. 2016; Perozo et al. 2002; Maingret et al. 2000; Bavi et al. 2017a, b). Together, both the movement of an MS channel caused by membrane stretching and insertion of amphipaths into one leaflet of the lipid bilayer can activate MS channels via changes of the transbilayer pressure profile asymmetry, which thus strongly suggests that the FFL paradigm of MS channel gating presents the general evolutionary conserved physicochemical principle underlying the MS channel–mediated mechanosensory transduction in cells of all living organisms.

## Piezo1 adventures with lipids

**Fig. 4:**
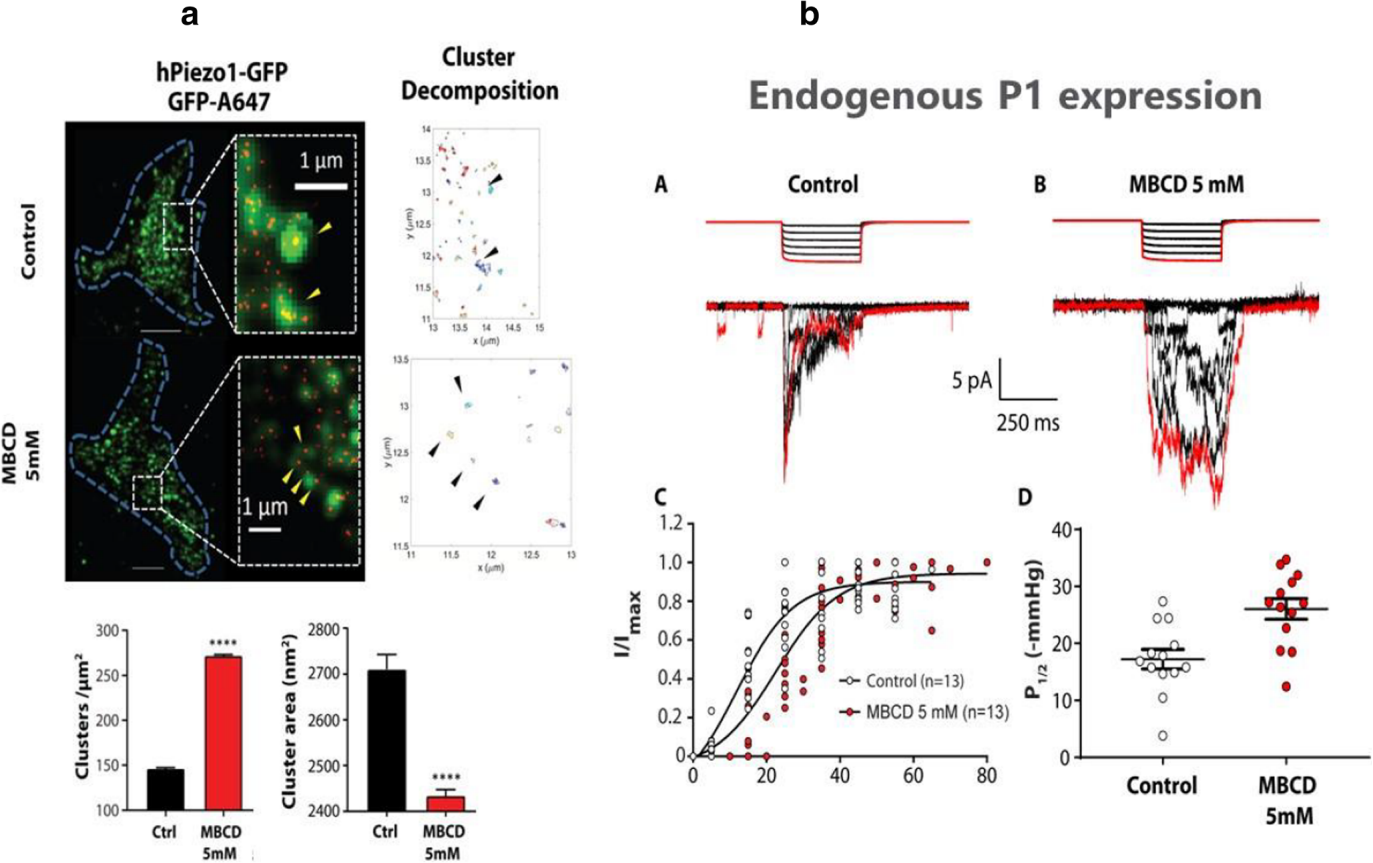
Cholesterol effect on Piezo1 clustering and channel kinetics. (**A**) Super-resolution microscopy shows the channels clustering together at the nanoscale (top). These clusters are dependent on the cholesterol content of the membrane (bottom). (**B**) Leaching cholesterol from the membrane changes sensitivity and gating kinetics. Under pressure application in a membrane patch (right control) the current decays as the channel inactivates. This behavior is lost if cholesterol is removed (top). Boltzmann distribution function showing dependence of Piezo1 currents on negative pressure (suction) applied to the cell-attached membrane patch of HEK293 cells. Piezo1 channels in cells treated with 5 mM MBCD exhibit reduced mechanosensitivity as indicated by a shallowed slope of the Boltzmann function (bottom left). The half activation pressure of Piezo1 channels increased significantly in membrane patches treated with MBCD (bottom right) (modified from Ridone et al. (2019))

Currently, little is known about Piezo1 plasma membrane localization and organization. However, what is known is that Piezo1 ion channels are inherently mechanosensitive and therefore, their interactions with membrane lipids are essential for their function (Cox et al. 2016a; Syeda et al. 2016). Indeed, cholesterol enrichment or depletion by methyl-β-cyclodextrin (MBCD) and disruption of membrane cholesterol organization by dynasore have recently been shown to affect Piezo1 response to mechanical force (Ridone et al. 2019). Electrophysiological recordings in the cell-attached configuration revealed that MBCD caused a rightward shift in the Piezo1 pressure-response curve, increased channel latency in response to mechanical stimuli, and markedly slowed channel inactivation (Fig. 4). The same effects were seen in native Piezo1 in N2A cells.

STORM super-resolution imaging revealed that at the nanoscale, Piezo1 channels in the membrane associate as clusters sensitive to membrane manipulation. Both cluster distribution and diffusion rates were affected by treatment with 5 mM MBCD. Supplementation of poly-unsaturated fatty acids appeared also to sensitize the Piezo1 response to applied pressure. Consequently, these results indicate that Piezo1 function is strongly dependent on the membrane mechanical properties and lateral organization of membrane cholesterol domains, referred to also as “lipid rafts” (Nicolson 2014), which coordinate the concerted channel activity. Lipid rafts are membrane microdomains composed of combinations of cholesterol, glycosphingolipids, and protein receptors (Nicolson 2014). Compared with the surrounding lipid bilayer, they are more ordered and tightly packed. Interestingly, cholesterol-enriched platforms such as lipid rafts are universally present in cellular force foci, which can be defined as points of force reception characteristic of specialized forms of mechanotransduction, including hearing and touch, as well as of more general cadherin foci and integrin foci (Ingber 2006). Lipid rafts thus could provide a hybrid mechanism between the FFL and FFF mechanisms of gating Piezo1 and other inherently mechanosensitive channels by mechanical force. Therefore, the “either-or-distinction” between the FFL and FFF paradigm seems superfluous.

In addition to cholesterol, phosphoinositides (e.g., PIP2) have been shown to be functionally relevant as well as closely related to mechanopathologies resulting from malfunction of Piezo1. During our studies of Piezo1 interactions with the surrounding lipid environment, we also found that not only PIP2 but also PIP1 and PIP3 were highly enriched around the protein (unpublished results). A binding site consisting of four lysines, K2166–K2169, in the human form of Piezo1 was identified around the channel pore domains. Notably, the four lysines are highly conserved in Piezo1 channel homologs. The removal of the four lysines in a deletion Δ4K mutant was reported to cause xerocytosis, a familial anemia characterized by a dehydrated form of red blood cells (Albuisson et al. 2013). Furthermore, in patch clamp experiments, this deletion mutation was shown to reduce channel inactivation significantly (unpublished results).

## Role of the cytoskeleton and extracellular matrix in Piezo1 channel gating

Together with MS channels, cytoskeleton (CSK) is another firmly established cellular mechanosensor^54^.

**Fig. 5:**
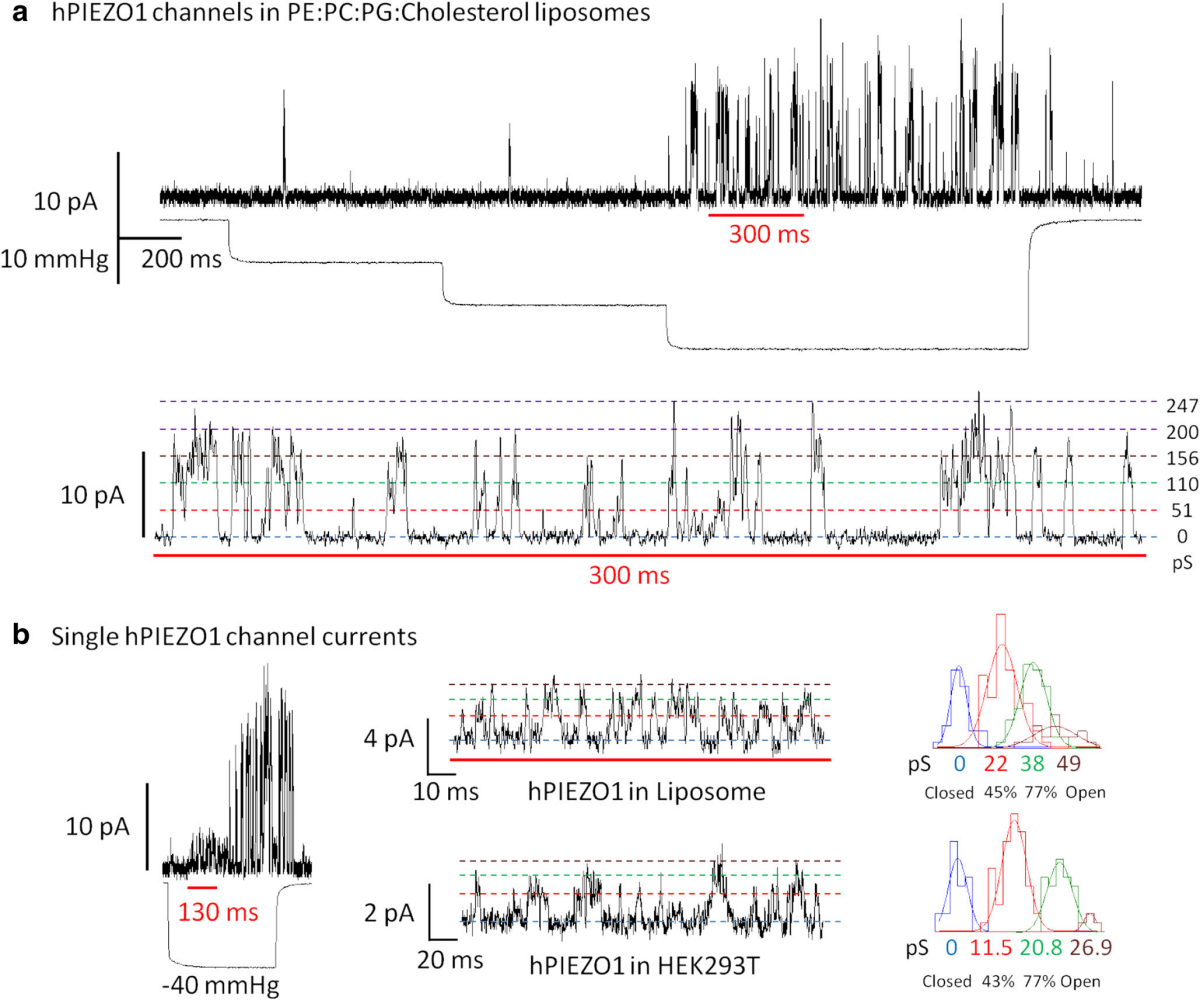
Mechanical activation of purified human Piezo1 (hPiezo1) reconstituted in artificial liposomes made of PE:PC:PG:Cholesterol. (**A**) Representative inside-out patch clamp recording of hPIEZO1 at + 65 mV pipette potential. Inward currents produced in response to 10 mmHg incremental steps in suction. Bottom: inset shows the concerted activity of up to 5 hPIEZO1 channels and the observed conductance values (pS) are indicated on the right. (**B**) Mechanical activation of single PIEZO1 channel recorded in artificial liposomes and summarized in a representative histogram of conductance values on the right-hand side. Bottom: single hPIEZO1 activity in HEK293T cells transfected with the same construct used for protein purification and representative histogram of single channel events (liposome recording solution: 200 mM KCl, 40 mM MgCl2, 5 mM HEPES, pH 7.4. HEK293 cell recoding solution: 140 mM NaCl, 3 mM KCl, 1 mM MgCl2, 1 mM CaCl2, 10 mM glucose, 10 mM HEPES, pH 7.4) (unpublished results)

Though similar to the bacterial MscL and MscS channels, Piezo1 also functions in reconstituted planar bilayers (Jaggers et al. 2019; Syeda et al. 2016), and critical role of lipids for the Piezo1 channel activity has clearly been established (Cox et al. 2016b). Recent experimental work conducted in our laboratory demonstrated how purified Piezo1 could be activated by applying pipette suction in patch clamp experiments on reconstituted proteoliposomes (Fig. 5) (unpublished results) similarly to MscL and MscS (Nomura et al. 2012).

This in no way precludes a role for the cytoskeleton in Piezo1 function (Gottlieb and Sachs 2012; Poole et al. 2014) because membrane forces are largely determined by the local arrangement of the cytoskeleton and extracellular matrix. Integrins, for example, form adhesions on the surface of mammalian cell membranes allowing mechanical stimuli to be focused on different CSK components, which directly or indirectly transmit these stimuli to membrane proteins, including ion channels (Wang et al. 1993). To fulfill this role, CSK is organized as “tensegrity” structures pre-stressing the cells to keep the cell shape stabilized by the network of opposing tension and compression components (Ingber 2006). As recently demonstrated for Piezo1 channels, which are gated according to the FFL paradigm (Syeda et al. 2016), the important function of the CSK and extracellular matrix (ECM) is therefore to mechanically protect the lipid bilayer by absorbing mechanical stresses (Cox et al. 2016a) and possibly also to alter the forces by modifying the time dependence of MS channel desensitization (Hamill and Martinac 2001). Furthermore, it has also been shown that the presence of some CSK proteins, such as STOML3 and tropomyosin 4.2 (Tpm4.2), apparently prestresses the cell membrane, which results in increased mechanosensitivity of Piezo1 to membrane tension (Cox et al. 2018). Alternatively, the presence of filamin A was shown to reduce the mechanosensitivity of the channel.

Moreover, an atomic force microscopy (AFM) study reported that mechanical stimulation of Piezo1 was dependent on the channel connections/interactions with ECM proteins (Gaub and Muller 2017). Piezo1 channels were relatively insensitive to mechanical stimuli pushing the cell membrane, whereas forces pulling the membrane could activate the channel effectively. The protein that was found to sensitize Piezo1 to pulling forces was identified as collagen IV, which is a component of the basal lamina forming a cohesive network and mechanical connection between cells. Consequently, not only the direct interaction between Piezo1 and ECM but also directionality of mechanical force can modulate Piezo1 sensitivity to mechanical stimuli.

## Piezo1 channel function in the (patho)physiology of mechanotransduction

The importance of the malfunctioning MS ion channels as contributors to pathology of a variety of hereditary diseases of previously unknown etiology has increasingly caught the attention of medical practitioners. This includes a variety of mechanochannelopathies where point mutations in an MS channel can be traced as the causative entity.

Despite their relatively recent discovery Piezo, ion channels have already been closely linked to many processes defining mechanotransduction processes in mammalian cells. Both Piezo1 and Piezo2 are widely expressed in the tissues of hollow organs such as the stomach, lungs, bladder, intestines, and endothelial cells lining the lumen of blood vessels (Martinac and Cox 2017). The central role they seem to play in mammalian organisms is underlined by the fact that global knockouts of Piezo1 in mice result in embryonic lethality (Li et al. 2014). Furthermore, gain-of-function (GOF) mutations in human Piezo1 cause hereditary xerocytosis (also known as dehydrated stomatocytosis), a familial anemia (Bae et al. 2013; Gottlieb and Sachs 2012), whereas loss-of-function (LOF) mutations cause generalized lymphatic dysplasia characterized by varying degrees of anemia (Fotiou et al. 2015; Lukacs et al. 2015). Both channelopathies suggest a central role that Piezo1 plays in erythrocyte volume control. As the research on MS ion channels continues progressing and the new knowledge of MS channel structure and function continues growing, new MS channel proteins may become discovered and new links between malfunctioning MS channels and related mechanopathophysiology can be found. Thus, our awareness of diseases linked to malfunctioning MS channels will increase and will make MS channels attractive targets for the development of new drugs to treat a variety of mechanochannelopathies in the future.

Studies on the effects of lipids such as cholesterol on PIEZO1 activity (Romero et al. 2019; Ridone et al. 2019) suggest that signal compartmentalization from specific membrane regions might explain how the conduction of such a ubiquitous ion such as Ca^2+^ could trigger very specific mechanosensitive responses. All regulatory modes described above (CSK, ECM, lipid rafts, protein-protein interactions, and channel clustering) ultimately fine-tune the sensitivity, magnitude, and duration of Piezo1 currents. Compartmentalization of mechanosensitive signaling facilitates an efficient and specific transduction process to achieve localized subcellular remodeling. Tensile forces are instantaneously transmitted across large distances and the remodeling action of the actin-severing machinery can impact mechanosensitivity and force-dependent signaling at locations far from the point of force detection (force foci) (Burridge and Guilluy 2016). The Piezo1 channel is in fact ideally placed in the cellular context to respond to both “Outside-In” (external stimuli) and “Inside-Out” (cell-generated) signaling (Kwong et al. 2003). This results in not only biochemical feedback loops (e.g., filamin A (Retailleau et al. 2015) and myosin II (Ellefsen et al. 2018)) but also genetic feedback loops that drive the overexpression of Piezo1 channels, as described in multiple cell types from animal models and in vitro (Satoh et al. 2006; Liu et al. 2018; Jones et al. 2018; Etem et al. 2018; Velasco-Estevez et al. 2018; Jin et al. 2015; Liang et al. 2017; Michishita et al. 2016). While biochemical feedbacks might impact the rearrangement events on the short time scales, the genetic feedbacks represent critically relevant mediators of pathology and cancer since they have a long-term impact on the activity of the many oncogenic pathways linked to Piezo1 (e.g., HIPPO, MAPK, ERK).

## Pharmacology of Piezo1 mechanosensitive channels

At present, there are only a few agents and compounds generally used for applications in MS channel research. They include the lanthanides Gd^3+^ and La^3+^, the aminoglycoside antibiotics such as streptomycin and gentamicin, and the GsMTx-4 peptide isolated from the toxin of the spider *Grammostola spatulata*, which are known to block most of the known MS channels (Martinac and Cox 2017; Hamill and McBride 1996). Another group of compounds, such as amphipaths chlorpromazine and LPC, have been shown to activate both prokaryotic and eukaryotic MS channels (Martinac and Cox 2017; Hamill and McBride 1996). In addition to these “typical” MS channel blockers and activators, there are more specific compounds affecting only a certain type of MS channels. For example, ruthenium red and Yoda1 block and activate Piezo1, respectively (Coste et al. 2012; Syeda et al. 2015). More recently, an analog of Yoda1, named Dooku1, was synthetized and shown to reversibly antagonize Yoda1-induced activation of Piezo1 by competing for a specific channel binding site (Evans et al. 2018). A comprehensive and up-to-date list of blocking and activating agents of various types of MS channels can be found in Martinac and Cox (2017).

## Conclusions

In this review, we attempted to illustrate generally as well as specifically on the example of Piezo1 channels what makes MS channels unique and why we believe it is important to study them and to understand their biophysical gating principles. These channels are the force-sensing molecules providing information about the outside world through direct contact cells have with their surroundings. Sometimes, this information may not be exactly transduced into a proper intracellular signaling due to aberrant channel function, as it is apparently the case in hereditary mechanopathologies we briefly reported here. Our knowledge of the structure and function of MS channels comes to the rescue by helping us to understand the correct etiology of these pathologies. This emphasizes the need for more knowledge and better understanding of the MS channel diversity and their equally diverse structure and function, which can only be achieved through continuous discoveries and painstaking research. Despite the progress that has been made over the past 30 odd years in this field, there are still many outstanding questions. Thus, we look forward to future developments that may open new avenues for the treatment of a whole host of mechanochannelopathies, which should enrich our knowledge and improve human health conditions.

## Glossary

**Amphipaths**: A term describing a chemical compound possessing both hydrophilic and lipophilic properties.
**Archaea**: A group of single-celled microorganisms, which like bacteria have no cell nucleus or any other organelles within their cells.
**GPCRs**: G protein-coupled receptors are cell surface receptors activated by different types of stimuli, including light and binding of peptides, proteins, and lipids. They form the largest and highly diverse group of membrane receptors in eukaryotic organisms.
**GsMTx-4**: One of the peptide toxins isolated from the venom of the Chilean rose tarantula spider *Grammostola spatulata* that is known to block mechanosensitive ion channels.
**HEK293 cells**: Human embryonic kidney 293 cells frequently used in cell biology and biotechnology for transfection and production of viral vectors, vaccines, and recombinant proteins.
**MscL**: Bacterial mechanosensitive channel of large conductance.
**MscS**: Bacterial mechanosensitive channel of small conductance.
**N2A cells**: Neuro2a cells are a fast-growing mouse neuroblastoma cell line.
**OSCA**: A new class of mechanosensitive calcium-permeable ion channels in plants that are activated by high osmotic shock.
**PE, PC, and PG**: Abbreviations for major phospholipid constituents of biological membranes: phosphatidylethanolamine, phosphatidylcholine, and phosphatidylglycerol, respectively.
**STOML3**: Stomatin-like protein-3, which controls cell membrane mechanics by binding cholesterol.
**STORM**: Stochastic optical reconstruction microscopy is a super-resolution imaging technique utilizing sequential activation and time-resolved localization of photo-switchable fluorophores to create high-resolution images.
**TREK-1, TREK-2, and TRAAK**: Mechanosensitive members of the two-pore-domain background potassium (K2P) channel protein family, which includes functionally distinct subgroups: TWIK, THIK, TASK TALK, TREK, and TRESK.
